# Evaluating the fermentation characteristics, bacterial community, and predicted functional profiles of native grass ensiled with different additives

**DOI:** 10.3389/fmicb.2022.1025536

**Published:** 2022-10-18

**Authors:** Shuai Du, Sihan You, Xiaowei Jiang, Yuyu Li, Ruifeng Wang, Gentu Ge, Yushan Jia

**Affiliations:** ^1^National Engineering Laboratory of Biological Feed Safety and Pollution Prevention and Control, Key Laboratory of Molecular Nutrition, Ministry of Education, Key Laboratory of Animal Nutrition and Feed, Ministry of Agriculture and Rural Affairs, Key Laboratory of Animal Nutrition and Feed Science of Zhejiang Province, Institute of Feed Science, Zhejiang University, Hangzhou, China; ^2^Key Laboratory of Forage Cultivation, Processing and High Efficient Utilization, Ministry of Agriculture, Resources and Environment, Inner Mongolia Agricultural University, Hohhot, China; ^3^Institute of Grassland Research, Chinese Academy of Agricultural Sciences, Hohhot, China; ^4^Inner Mongolia Yihelvjin Agricultural Development Co., Ltd., Chifeng, China

**Keywords:** lactic acid bacteria, fermentation quality, bacterial community, silage, native grass

## Abstract

Bioaugmentation of native grass ensiling with *Lactobacillus plantarum* or *Lactobacillus buchneri* or *Pediococcus pentosaceus* on the ensiling performance and bacterial community was investigated after 30 days of the fermentation process. The native grass was inoculated with distilled water, *Lactobacillus plantarum, Lactobacillus plantarum*, and *Lactobacillus buchneri*, and *Lactobacillus plantarum, Lactobacillus buchneri*, and *Pediococcus pentosaceus* as the CON treatment, T1 treatment, T2 treatment, and T3 treatment, respectively. The addition of lactic acid bacteria was added at a total of 1 × 10^6^ colony-forming unit/g of fresh weight. As expected, the markedly (*p* < 0.05) lower water-soluble carbohydrate content was tested in the T2 and T3 treatments compared to the CON and T1 treatments. Compared to the CON and T1 treatment, significantly (*p* < 0.05) higher crude protein content, and lower acid detergent fiber and neutral detergent fiber contents were found in the T2 and T3 treatments. Compared to the CON treatment, the pH significantly (*p* < 0.05) decreased in the lactic acid bacteria (LAB) inoculated silage, and the lowest pH was measured in the T3 treatment. Similarly, significantly higher lactic acid and acetic acid contents were also found in the T3 treatment compared to those in other treatments. After 30 days of ensiling, the Shannon and Chao1 indexes in silages decreased compared to that in the fresh materials (FMs). The principal coordinate analysis indicated that both FM and silage were distinctly separated in each treatment with no interactions on the confidence ellipse (*R* = 0.8933, *p* = 0.001). At the phylum level, the dominant phylum was shifted from Proteobacteria to Firmicutes after the fermentation process. Interestingly, *Weissella* dominated the fermentation in the CON treatment and *Lactobacillus* dominated the fermentation in all inoculated LAB silages at the genus level. Results of functional prediction analyses showed that the metabolism of amino acid, cofactors, and vitamins, and membrane transport was reduced, while the metabolism of nucleotide and majority carbohydrates was increased after ensiling. The complex LAB (*Lactobacillus plantarum, Lactobacillus buchneri*, and *Pediococcus pentosaceus*) exhibited the potential possibility to decrease pH and enhance the relative abundance of LAB in response to obtaining high-quality silage by the synergistic effects. These results suggested that the complex LAB could improve the ensiling performance of native grass silage, and lay a theoretical basis for inoculant application in native grass.

## Introduction

The availability of feed in terms of quality, quantity, and continuity directly influences livestock productivity (Tahuk et al., 2021). Native grass provides feed for a large number of ruminants to meet the demand for meat and milk for consumers. Nevertheless, the quality and quantity of native grass are volatile because of seasonal changes (Tahuk et al., 2021; Du et al., 2022). The native grass production will increase during the summer, which leads to a positive effect on increasing biomass production, whereas, in the winter, low quality and feed deficiency constrained the continuous development of animal husbandry (Zhou et al., 2022).

Ensiling has received increased attention as a traditional, important, and reliable forages and grass preservation technique to provide a continuous supply for animal husbandry development throughout the year, particularly in developed countries (Zhou et al., 2019; Lin et al., 2021). Ensiling is a complex biochemistry process that was determined by several factors, including temperature, moisture, raw materials nutritional compositions, harvest time, raw materials length, pack density, the microbiome in raw materials, and others (Puntillo et al., 2022). In particular, the preservation of silage from forages and grasses depends on the microbial ecological diversity and the epiphytic lactic acid bacteria (LAB) play a determining role in the conservation of silage with high quality. The epiphytic LAB can be found ranging from 10^1^ to 10^7^ cfu/g^−1^ of raw material and the abundance may lead to undesirable fermentation when the epiphytic LAB was lower than 10^5^ cfu/g^−1^ of raw material (Cai et al., 1999). Different additives have been used to improve ensiling performance and preserve the nutrients in silage, among which LAB is one of the most current alternatives because it could improve the fermentation quality and prolong the storage of raw materials (Puntillo et al., 2022). The LAB inoculants could rapidly dominate and overcome the complex microbiome in the raw materials and novel strains have been isolated and identified from various materials and silages as new silage inoculants (Carvalho et al., 2021). It is difficult to directly produce high-quality native grass silage because of the lower moisture and water-soluble carbohydrate (WSC) contents, and lower LAB population (You et al., 2021a). A previous study indicated that the LAB could improve the fermentation quality of native grass silage, especially *Lactobacillus plantarum* (You et al., 2021a). Previously reports also have indicated that the homofermentative LAB could accelerate the fermentation process by utilizing the glucose to produce lactic acid (LA) through the Embden–Meyerhof pathway, such as the certain strains *Lactobacillus plantarum, Pediococcus acidilactici*, and *Pediococcus pentosaceus*, whereas, the heterofermentative LAB were also widely used for producing a mixture of LA, acetic acid (AA), and ethanol *via* the phosphoketolase pathway by improving the aerobic stability, such as the certain strains *Lactobacillus buchneri* (Gallagher et al., 2018; Alhaag et al., 2019; Cao et al., 2021; Silva et al., 2022). However, due to the diversity of, not all LAB inoculants could significantly improve silage, making it impossible to create universal LAB-based products (Fijalkowska et al., 2020; Cao et al., 2021).

Previous reports also found that the synergistic effects determine the final community structure and functions when the first species arrive (Cheong et al., 2021; Debray et al., 2021). Additionally, within specific niches, microbe–microbe interactions can play a critical role in driving community structures and functional properties (Cheong et al., 2021). Nevertheless, there is less research on how priority effects influence bacterial community structure and functional profiles in native grass inoculated with various LAB inoculants. As a result, the present study aimed to determine the following: the bioaugmentation efficacy of various LAB additives on ensiling performance in terms of fermentation characteristics, fermentation characteristics, and bacterial community, the correlations between ensiling performance and bacterial community in native grass silage and the functional profiles.

## Materials and methods

### Substrate and silage preparation

The native grass was collected in the typical steppe flora of Bairin Left Banner, Inner Mongolian Plateau, China. *Stipa gigantea* L. and *Leymus chinensis* (Trin.) Tzvel. as the dominant species in this grassland. To get high-quality native grass and silage, the native grass was harvested at the 5 cm cutting height at the milk stage of the dominant species (You et al., 2021b). The native grass was inoculated with *Lactobacillus plantarum* (Chikuso-1, Snow Brand Seed Co., Ltd, Sapporo, Japan) at 1 × 10^6^ colony-forming unit/g fresh matter as the T1 treatment; the native grass was inoculated with a mixture of *Lactobacillus plantarum* and *Lactobacillus buchneri* (Sci-plus Biotech. Co, Ltd, Inner Mongolia, China) at 1 × 10^6^ colony-forming unit/g fresh matter as the T2 treatment and the native grass was inoculated with a mixture of *Lactobacillus plantarum, Lactobacillus buchneri*, and *Pediococcus pentosaceus* (Lactosan GmbH and Co, KG, Austria) at 1 × 10^6^ colony-forming unit/g fresh matter as the T3 treatment, the native grass ensiling directly with the same volume deionized water as the control group (CON). The native grass was chopped into 30 mm size by a forage cuter (Fulida Tool Co., Ltd., Linyi, China) and then taken into the laboratory immediately. The native grass treated without or with LAB inoculants was transferred into vacuum-sealing polyethylene plastic bags (260 mm × 180 mm) and vacuum-sealed. Each group was ensiled with three replicates with about 250 g of forage per bag. The native grass silage bags were stored at room temperature (25~27°C) and sampled after 30 days of the fermentation period. The chemical compositions, fermentation quality, and bacterial community were analyzed after the silage bags ensiling for 30 days.

### Fermentation characteristics and chemical compositions analyses

For chemical composition parameters, the fresh weight (FW) and native grass silage were blended uniformly before sampling and the samples were put into envelopes. The samples were measured for dry matter (DM) content in an oven for 72 h at 65°C and then passed a 1-mm screen (FW100, Taisite Instrument Co., Ltd., Tianjin, China) for subsequent analysis. The anthrone method was used to determine the WSC content (Thomas, 1977). The ash and crude protein (CP) contents were analyzed based on the methods of the Association of Official Analytical Chemists (methods: 2001.11; AOAC, 2005). The acid detergent (ADF) and neutral fiber (NDF) contents were tested with an ANKOM A200i Fiber Analyzer (ANKOM Technology, Macedon, NY, USA) and were described exclusively as residual ash (Van Soest et al., 1991; Li et al., 2020). Ten grams of silage samples were mixed with 90 ml of deionized water to derive extract at 4°C fridge for 24 h. Then, the extracts underwent filtration through a four-layer cheesecloth for analyzing fermentation characteristics. The pH value of the filtrate was tested by a glass-electrode pH meter. The ammonia nitrogen (NH_3_-N) content was analyzed with the previous report (Broderick and Kang, 1980). Organic acid contents of the filtrate were analyzed by high-performance liquid chromatography (HPLC) with a UV detector (210 nm) and 3 mmol/L of HClO_4_ was the mobile phase at a flow rate of 1.0 mL min^−1^ at 50°C (You et al., 2021b). The microbial population in the fresh materials (FMs) was counted by the plate count method and expressed on colony-forming units (cfu)/g of FW. The numbers of LAB and coliform bacteria were counted on de Man, Rogosa, Sharpe agar (Difco Laboratories, Detroit, MI, USA) and blue light broth agar (Nissui Ltd., Tokyo, Japan) incubated at 30°C for 48 h, the numbers of mold and yeast, and aerobic bacteria were counted on potato dextrose agar (Nissui Ltd., Tokyo, Japan) and nutrient agar Nissui Ltd., Tokyo, Japan) incubated at 30°C for 24 h, respectively (You et al., 2021b).

### DNA extraction, PCR amplicon, and sequencing

Before analyzing the microbiome of the native grass silage, all samples were stored at −80°C before the extraction of DNA. The microbial DNA of FM and silage was extracted following the manufacturer's protocols with the HiPure Stool DNA Kits (Magen, Guangzhou, China). Amplicons spanning the V3–V4 hypervariable regions of the 16S rRNA gene were produced using primers 341F (5′-CCTACGGGNGGCWGCAG-3′) and 805R (5′-GACTACHVGGGTATCTAATCC-3′) to determine the bacterial community (Logue et al., 2016). The sequences of these samples have been uploaded into the public database with the accession number PRJNA871931.

### Microbial community analyses

The Flash (v1.2.8) was used to assign, truncate, and merge the paired-end reads (Tanja and Salzberg, 2011). Operational taxonomic units (OTUs) with a 97% similarity cutoff (Liu et al., 2019) were clustered by UPARSE (version 7.1, http://drive5.com/uparse/), and the high-quality sequences higher than 97% similarity were put into the same OTU (Rognes et al., 2016). The OTUs were classified using the Silva database (https://www.arbsilva.de/) with a confidence threshold of 70%, and the false discovery rate-adjusted Kruskal–Wallis multiple comparisons (*q* < 0.05) were used to detect bacterial community at the phylum and genus levels (Omontese et al., 2022), and permutational multivariate analysis of variance test was used to analyze the significant difference (Chambers and Hastie, 1992). The Venn diagram was constructed by R (v 1.6.2) according to the unique and common OTUs. The alpha diversity and Good's coverage were calculated in QIIME (version 1.9.1; Caporaso et al., 2010). The principal coordinates analysis (PCoA) was generated based on Bray–Curtis in the R package (2.5.3). The bacterial abundance and community composition were performed with Krona (version 2.6) and R package (version 2.2.1), respectively (Ondov et al., 2011; Wickham, 2011). The pheatmap package was used to display the heatmap of genus abundance (version 1.0.12; Kolde and Kolde, 2015). Spearman correlation analysis of species was calculated in the R package (version 1.8.4; Revelle and Revelle, 2015). The linear discriminant analysis (LDA) effect size (LEfSe) analyses were conducted *via* an online tool (https://www.omicstudio.cn/tool/60), and the LDA score > 4 and *p* < 0.05 were selected as the threshold.

### Statistical analysis

The analyzed data of chemical compositions (DM, CP, WSC, ADF, and NDF contents) and fermentation characteristics (pH, LA, AA, PA, BA, and NH_3_-N parameters) were expressed on the mean ± standard error of means of three replicates. The effects of additives on the silage quality were evaluated with SAS 9.0 (SAS Institute, 2007 Cary, NC, USA). All the measured data were analyzed with the additive effect by the general linear models (GLMs): Y_ij_ = μ + α_i_ + ε_ij_, where, μ is the overall mean, α_i_ is the additive effect, and ε_ij_ is the residual error (Ren et al., 2021).

## Results

### Chemical and microbial compositions of native grass

The chemical compositions and microbial population of the FM before ensiling are shown in Table 1. The DM content was 51.27% of the raw materials. The concentrations of WSC, CP, NDF, and ADF of native grass were 4.45, 11.29, 70.30, and 38.54% of DM, respectively. The native grass contained low desirable LAB (3.52 log cfu/g of FW) and high coliform bacteria (6.58 log cfu/g of FW) counts. The numbers of aerobic bacteria and yeasts were 6.29 and 6.33 log cfu/g of FW, respectively. Mold was not detected.

**Table 1:** Chemical and microbial compositions of substrates prior to ensiling.

**Items**	**Native grass**	**SEM**
Dry matter (%)	51.27	0.55
Water-soluble carbohydrates (% DM)	4.45	0.07
Crude protein (% DM)	11.59	0.15
Neutral detergent fiber (% DM)	70.30	0.19
Acid detergent fiber (% DM)	38.54	0.66
LAB (log_10_ cfu/g FW)	3.52	0.39
Aerobic bacteria (log_10_ cfu/g FW)	6.29	0.06
Yeasts (log_10_ cfu/g FW)	6.33	0.23
Coliform bacteria (log_10_ cfu/g FW)	6.58	0.28
Mold (log_10_ cfu/g FW)	ND	

DM, dry matter; FW, fresh weight; lg, denary logarithm of the numbers; cfu, colony-forming units. LAB, lactic acid bacteria. ND, not detected. SEM, standard error of mean.

### Ensiling performance of native grass without or with LAB inoculation

The fermentation quality and nutritional profiles of native grass ensiled with various LAB are shown in Table 2. As expected, the markedly (*p* < 0.05) lower WSC content was tested in the T2 and T3 treatments compared to the CON and T1 treatments, and no significant difference was found between the CON and T1 treatments. Interestingly, no significant difference was found in the DM content among these treatments. Additionally, compared to the CON and T1 treatments, significantly (*p* < 0.05) higher CP content, and lower ADF and NDF contents were found in the T2 and T3 treatments. After 30 days of ensiling, compared to the CON treatment, the pH was decreased in the LAB-inoculated silage, and the lowest pH was measured in the T3 treatment. Similarly, the highest LA and AA contents were also found in the T3 treatment compared to those in other treatments. No significant difference was analyzed in the propionic acid (PA) and butyric acid (BA) concentrations among these treatments.

**Table 2:** Fermentation characteristics of native grass ensiled with various lactic acid bacteria.

**Item**	**CON**	**T1**	**T2**	**T3**	**SEM**	***p*-value**
pH	5.94^a^	5.11^b^	5.07^b^	4.68^c^	0.14	< 0.0001
NH_3_-N (g/kg DM)	7.49^a^	4.28^b^	3.62^b^	2.50^c^	0.57	< 0.0001
LA (g/kg DM)	23.20^c^	32.80^b^	33.48^b^	38.51^a^	1.60	< 0.0001
AA (g/kg DM)	64.32^b^	66.70^b^	76.23^ab^	87.20^a^	3.22	0.02
PA (g/kg DM)	1.44	1.52	1.77	1.89	0.09	0.28
BA (g/kg DM)	0.80	0.73	0.63	0.66	0.03	0.17
DM (%)	47.29	46.18	46.60	46.32	0.46	0.53
WSC (g/kg DM)	3.44^a^	3.34^a^	3.07^b^	2.86^c^	0.07	< 0.0001
CP (g/kg DM)	10.18^c^	10.59^b^	10.34b^c^	11.24^a^	0.13	< 0.0001
ADF (g/kg DM)	35.66^a^	35.34^ab^	33.67^bc^	33.30^c^	0.38	0.03
NDF (g/kg DM)	67.49^a^	66.69^ab^	64.42^b^	64.38^b^	0.53	0.04

DM, dry matter; WSC, water-soluble carbohydrates; CP, crude protein; ADF, acid detergent fiber, NDF, neutral detergent fiber; NH_3_-N, ammonia nitrogen; LA, lactic acid; AA, acetic acid; PA, propionic acid; BA, butyric acid. CON, Samples without inoculants; T1, native grass inoculated with *Lactobacillus plantarum*; T2, native grass inoculated with *Lactobacillus plantarum* and *Lactobacillus buchneri*; T3, native grass inoculated with *Lactobacillus plantarum, Lactobacillus buchneri*, and *Pediococcus pentosaceus*. SEM, standard error of mean. ^a, b, c^ significant differences at *p* < 0.05 level.

### Bacterial diversity of native grass inoculated without or with LAB

**Figure 1:**
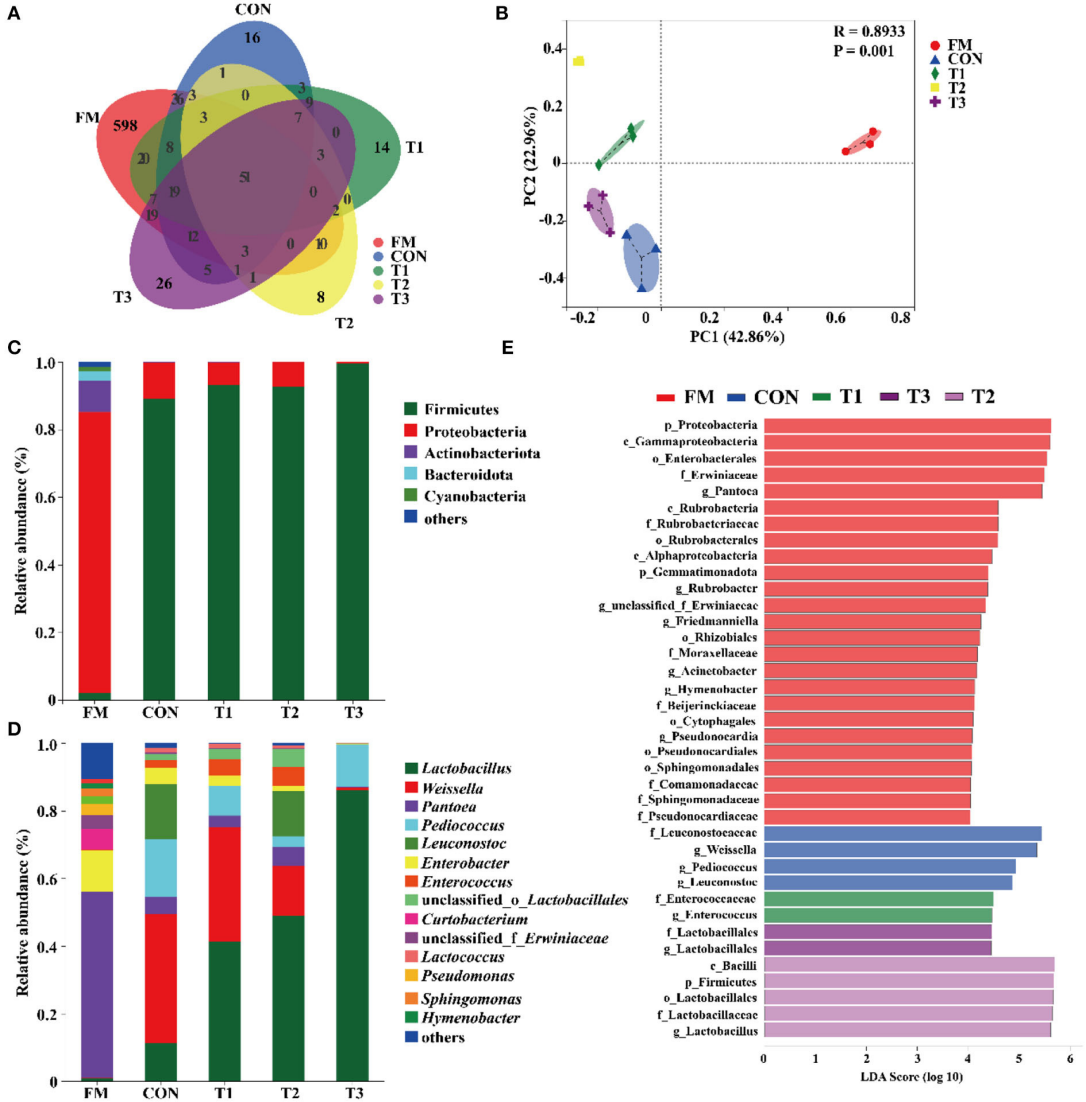
Bacterial community diversities and dissimilarities of native grass silage. FM, fresh material; CON, Samples without inoculants; T1, native grass inoculated with *Lactobacillus plantarum*; T2, native grass inoculated with *Lactobacillus plantarum* and *Lactobacillus buchneri*; T3, native grass inoculated with *Lactobacillus plantarum, Lactobacillus buchneri*, and *Pediococcus pentosaceus*. **(A)** Venn diagram representing the unique and common OTUs. **(B)** The bacterial community dissimilarities in different treatments were calculated by Bray–Curtis with coordinates calculated by principal coordinates analysis. **(C,D)** Relative abundances of native grass silage bacterial phylum and genus across different treatments. **(E)** Differences of bacterial taxa in native grass silage with different treatments.

The diversity of bacteria in native grass silage is shown in Table 3. According to the 16S rRNA amplicon sequencing of native grass materials and silage bacteria, an average of 60, 847 sequence numbers was obtained from each sample (data are not shown) and no significant difference was found in sequence numbers among these samples. After 30 days of ensiling, the diversity (Shannon) and richness (Chao1) in silages were decreased compared to that in the FM. A Venn diagram was constructed to depict similar and overlapping OTUs among all raw materials and silage (Figure 1A). As displayed, 51 OTUs were shared by the FM, CON, T1, T2, and T3 treatments, and the unique OTUs among these treatments were 598, 16, 14, 8, and 26, respectively. As indicated in the PCoA plot (Figure 1B; *R* = 0.8933, *p* = 0.001), both fresh native grass and silage were separated in each treatment with no interactions on the confidence ellipse. Figure 1C shows the abundance of the phyla in the raw materials and silages after 30 days of ensiling. Proteobacteria dominated the community at the phylum level in the fresh native grass. After the fermentation process, Firmicutes dramatically increased and became the most abundant phylum in native grass silage. Figure 1C shows the community structures in the FM and silages after 30 days of ensiling at the genus level. In the FM, the most abundant genus was *Pantoea*, followed by *Enterobacter* and *Curtobacterium*. Interestingly, the bacterial community structures in native grass inoculated without or with LAB were diverse. The genus, *Weissella* plays the important role in the CON group, and the genus *Lactobacillus* dominated the fermentation in all LAB-inoculated silage. Differences of bacterial taxa in native grass silage with different treatments were performed by LEfSe analyses (Figure 1E). As described, the phylum Proteobacteria and genus *Pantoea* were significantly (*p* < 0.05) enriched in the FM compared to these in the silage, and the phylum Firmicutes was markedly (*p* < 0.05) enriched in the T3 treatment than that in the fresh native grass and other treatments. The genus *Weissella* was significantly (*p* < 0.05) concentrated in the CON and T1 treatments compared to that in the T2 and T3 treatments. Additionally, compared to the CON and T1 treatments, the genus *Lactobacillus* was dramatically (*p* < 0.05) enriched in the T2 and T3 treatments.

**Table 3:** Diversity indices of the bacterial community of materials or silage of native grass.

**Item**	**FM**	**CON**	**T1**	**T2**	**T3**	**SEM**	***p*-value**
No. of Sequence	58191	56301	55838	66705	67203	2706	0.54
Shannon index	0.61^a^	0.35^ab^	0.23^ab^	0.04^b^	0.25^ab^	0.07	0.08
Chao1 value	17.00^a^	9.11^b^	8.25^b^	10.00^ab^	7.83^b^	1.23	0.08
Coverage	0.99	0.99	0.99	0.99	0.99	< 0.0001	0.35

CON, Samples without inoculants; FM, fresh material; T1, native grass inoculated with *Lactobacillus plantarum*; T2, native grass inoculated with *Lactobacillus plantarum* and *Lactobacillus buchneri*; T3, native grass inoculated with *Lactobacillus plantarum, Lactobacillus buchneri*, and *Pediococcus pentosaceus*. SEM, standard error of mean. ^a, b^ significant differences at *p* < 0.05 level.

### Correlations between the bacterial community and ensiling performance in native grass inoculated without or with LAB

**Figure 2:**
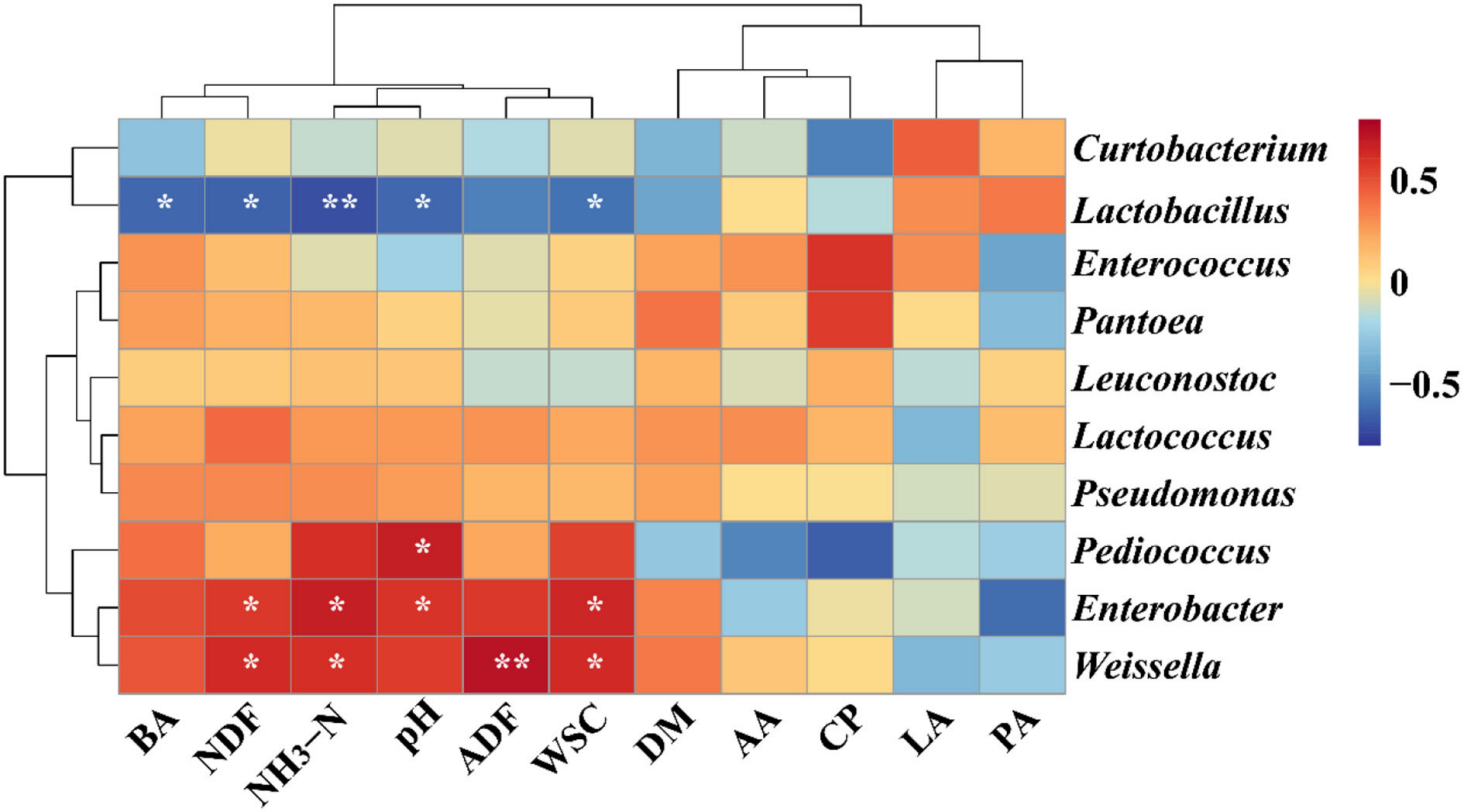
Correlation analysis between genera (top 10 significant genera) and ensiling performance of native grass silage inoculated without or with lactic acid bacteria. *Significant correlation at *p* < 0.05 level, **Significant correlation at *p* < 0.01 level. DM, dry matter; WSC, water-soluble carbohydrates; CP, crude protein; ADF, acid detergent fiber; NDF, neutral detergent fiber; NH_3_-N, ammonia nitrogen; LA, lactic acid; AA, acetic acid; PA, propionic acid; BA, butyric acid.

In the present study, the heatmap was used to evaluate the correlations between the bacterial genus (Top 10) and chemical constituents/fermentation profile based on the Spearman analysis in Figure 2. The BA, NDF, NH_3_-N and WSC contents, and pH were significantly associated with *Lactobacillus* (BA: rho = −0.631, *p* = 0.028; NDF: rho = −0.650, *p* = 0.022; NH_3_-N: rho = −0.713, *p* < 0.01; WSC: rho = −0.592, *p* = 0.04; pH: rho = −0.630; *p* = 0.03), whereas, the genus *Pediococcus* was positively associated with CP content (rho = 0.681; *p* = 0.02). The NDF, NH_3_-N, and WSC contents were also observed to have significantly positive connections with the genus *Enterobacter* (NDF: rho = 0.584, *p* = 0.05; NH_3_-N: rho = 0.674, *p* = 0.02; WSC: rho = 0.652, *p* = 0.02) and *Weissella* (NDF: rho = 0.625, *p* = 0.03; NH_3_-N: rho = 0.608, *p* = 0.04; WSC: rho = 0.627, *p* = 0.03).

### Predicted functions and pathways of bacterial community in native grass silage

**Figure 3:**
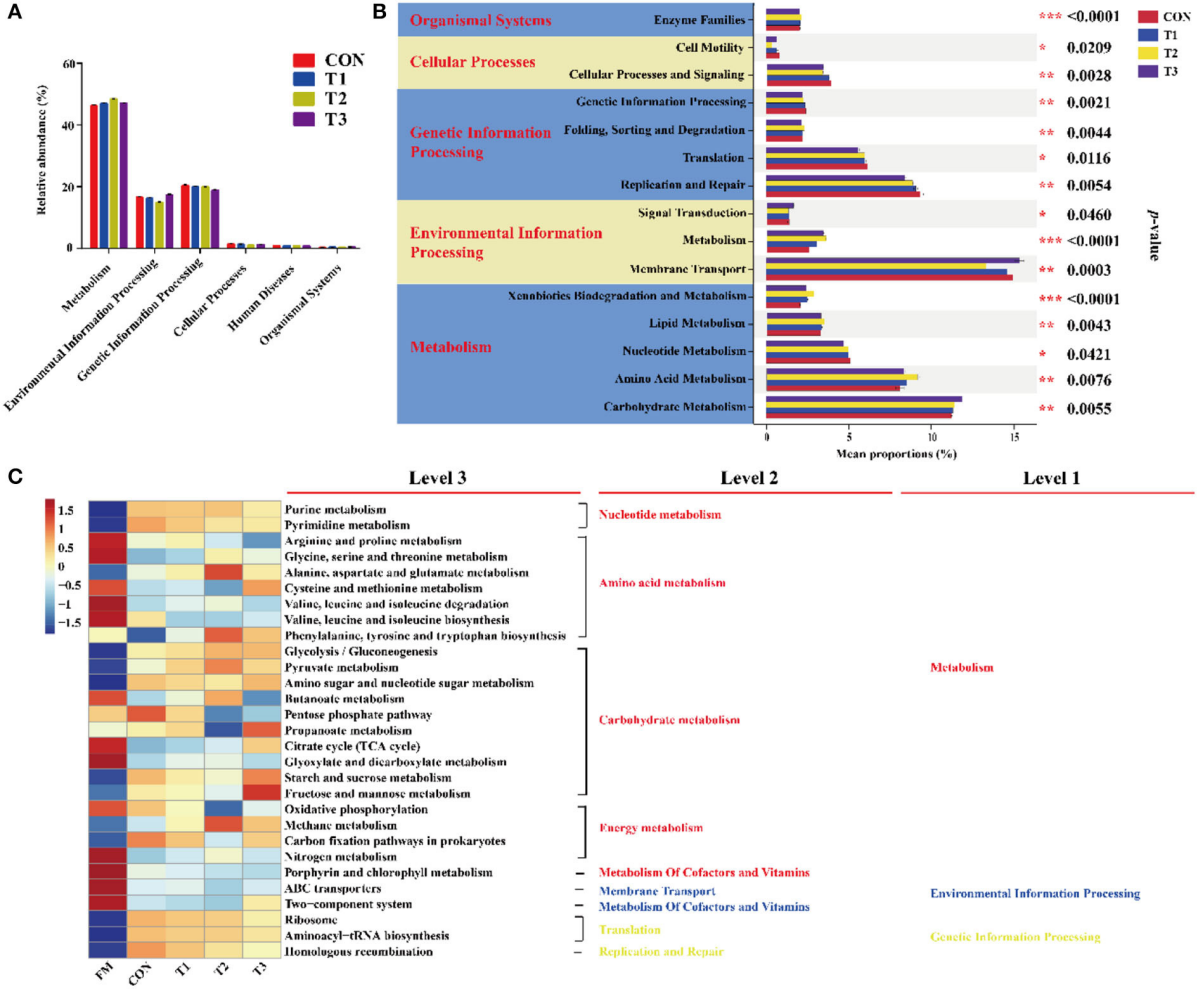
Dynamics of bacterial functional profiles in different treatments analyzed by PICRUSt (*n* = 3). **(A)** Level 1 metabolic pathway. **(B)** Level 2 KEGG ortholog functional predictions of the relative abundances with significant differences in the top 20 metabolic functions. **(C)** Level 3 KEGG ortholog functional predictions of the relative abundances of the top 30 metabolic functions. FM, fresh materials; CON, Samples without inoculants; FM, fresh material; T1, native grass inoculated with *Lactobacillus plantarum*; T2, native grass inoculated with *Lactobacillus plantarum* and *Lactobacillus buchneri*; T3, native grass inoculated with *Lactobacillus plantarum, Lactobacillus buchneri*, and *Pediococcus pentosaceus*.de. * Indicates significant at *p* < 0.05, ** indicates significant at *p* < 0.01 and *** indicates significant at *p* < 0.001.

In the current study, the predicted function of bacterial communities was performed by the PICRUSt (Figure 3). As shown in Figure 3A, the abundance of “Metabolism” was more than 40% and was markedly higher than other pathways, followed by environmental information processing and genetic information processing. The top 20 with significant differences in metabolic function are shown in Figure 3B. The proportions of membrane transport, carbohydrate metabolism, and amino acid metabolism were much higher than the other pathways. The carbohydrate metabolism was significantly (*p* < 0.05) inhibited in LAB-inoculated silage, especially in the T3 treatment, whereas the proportions of amino acid metabolism were significantly (*p* < 0.05) increased in the T1 treatment compared to the other treatments. Interestingly, compared to the CON group, the proportion of membrane transport was markedly in the LAB-inoculated silage. As shown in Figure 3C, the metabolic functions of the bacterial community were diverse in FM and silage. Results of functional prediction analyses showed that the metabolism of amino acid, cofactors, vitamins, and membrane transport was reduced, while the metabolism of nucleotide and the majority of carbohydrates was increased after ensiling. The carbohydrate metabolism (TCA cycle, starch and sucrose metabolism, and fructose and mannose metabolism) and amino acid metabolism (cysteine and methionine metabolism) were enriched in the T3 treatment compared to that in the CON group.

## Discussion

Ensiling is a useful method to prolong the supply of feed to ruminants. The LAB inoculants, including *L. buchneri, L. plantarum*, and *P. pentosaceus* are widely used worldwide in silages. On the one hand, the chemical compositions in the forages and grass can be effectively preserved by anaerobic fermentation; on the other hand, the microorganisms also produce organic acid (mainly LA and AA) that can improve the nutritional structure during the fermentation process (Lin et al., 2021). Unfortunately, the effective LAB inoculant is limited to improving the ensiling performance on native grass, and the complex LAB on native grass silage remains largely unknown. Here, the multiple fermentation characteristic analyses were combined with the16S rRNA sequencing to reveal the changes in native grass inoculated without or with various LAB inoculants during anaerobic fermentation. This is the first tentative model for the integrative analysis of the bacteria to respond to the anaerobic bioaugmentation of native grass ensiling with various LAB inoculants.

### Chemical and microbial compositions of native grass

In the present study, the ADF and NDF contents were higher, and the CP content was lower compared to the previous report by You et al. (2021b), which could be contributed to the internal and external factors, including native grass community composition, harvest time and environment (Wang et al., 2020). The requirement of WSC content of good-quality silages is more than 5% DM (Amer et al., 2012), and the WSC content (4.45%) in the present study might meet the requirement. Moreover, the fermentation process was determined by the number of LAB, and the minimum requirement for the number of LAB in fresh materials should be higher than 5.0 log cfu/g FW (Cai et al., 1999). In the present study, the lower LAB (3.52 log cfu/g FW) counts and higher harmful microorganisms (more than 5.0 log cfu/g FW) were also found, which led to undesirable fermentation and end products. Therefore, it is necessary to add and reveal the role of LAB how to determine the silage fermentation and the shift of bacteria.

### Ensiling performance of native grass silage

The LA content plays a determining role in dropping pH in silage and the desirable pH is ~3.8–4.2 for well-preserved silage. Nevertheless, the pH in all native grass silages was higher than 4.20 after 30 days of the fermentation process, which is in disagreement with the previous report that the pH (< 4.20) of native grass with 37.72% DM content ensiled with LAB additives after 30 days of fermentation. These differences could be explained by the DM content. Compared to the CON treatment, the LAB-inoculated silage decreased the pH value and the lowest pH was found in the T3 treatment, which could be contributed to the LAB additives improving the accumulation of LA and AA by Embden–Meyerhof pathway, phosphoketolase pathway, and pentose phosphate pathway for homofermentative LAB and pentose phosphate pathway for the homofermentative LAB by utilizing the WSC content (Ganzle, 2015; Muck et al., 2018; Valk et al., 2020; Lee et al., 2021). Moreover, the T3 treatment inoculated with three species of LAB could produce an acidic environment that was more beneficial for the LAB by the synergistic effects, and the WSC content was continuously utilized by the species of LAB (Cai et al., 1999; Svoboda et al., 2018). Therefore, lower DM and pH, and higher LA and AA contents were found in the T3 treatment, especially, the lowest WSC content was observed in the T3 treatment, followed by the T2 and T1 treatments. The PA and BA contents are undesirable fermentation end products in silages because the production pathway is an energy-waste metabolism (Dong et al., 2022). The little production of PA and BA contents indicated that extensive secondary fermentation did not occur throughout the whole fermentation process (Dong et al., 2022). The NH_3_-N content is negatively associated with the CP content. The plant and microbial enzymes degraded the protein into non-protein fractions throughout the fermentation process, including NH_3_, NH_3_-N, free amino acids, and peptides, by the proteolysis (Dong et al., 2022), reflecting the protein degradation throughout the ensiling period (Kung et al., 2018). Furthermore, the lower NH_3_-N and higher CP contents in the T3 treatment indicated that the forage protein was well-preserved after the fermentation process, which could have contributed to the lower pH, which could have inhibited the growth and metabolism of undesirable microorganisms, such as *Clostridium* (Kung et al., 2018; You et al., 2021b). At the same time, the digestible cell wall was broken down under hydrolytic activities, including microbial activities, enzymatic, and acidolysis when the silage is made (Zhao et al., 2018). Consequently, lower ADF and NDF contents were observed in the T3 treatment.

### Bacterial diversity of native grass silage

The bacterial diversity and compositions in native grass silage were revealed by 16S rRNA sequencing. The variances of the bacterial community were performed by alpha diversity. The coverage in all samples was more than 0.99 (Table 3), suggesting that the depth of 16S rRNA sequencing had adequately reasonable to reflect the profile of the bacterial community (Ren et al., 2021). In the current study, the OTUs diversity and richness were decreased after ensiling compared to that in the FM (Table 3 and Figure 1A), which is in agreement with the previous reports that the decreased alpha diversity was observed because the undesirable microorganisms were inhibited by pH and gradually replaced by LAB (Xu et al., 2020; Ren et al., 2021). The PCoA plot clearly illustrates the variance of the bacterial community structures by the fermentation-additives-based separation of the treatments, suggesting that the additives had remarkable effects on the bacterial community structures of native grass silage. The predominant bacterial phylum, Proteobacteria, was found in FM, and the, most dominant bacterial phylum, Firmicutes, was found across all silage samples. In the current study, the dominant phylum was Proteobacteria and the primary genus was *Pantoea, Pseudomonas*, and *Sphingomonas* in FM, which is similar to the previous results that *Pantoea, Pseudomonas*, and *Sphingomonas* were the dominant genus in the raw materials (Ogunade et al., 2018; Romero et al., 2021; Long et al., 2022). Moreover, Firmicutes become the predominant phylum after the fermentation process (Yuan et al., 2020; Long et al., 2022). The shift from Proteobacteria to Firmicutes could be contributed to the microorganisms belonging to Firmicutes can thrive under low pH and anaerobic conditions (Wang et al., 2018; Dong et al., 2019). After ensiling, the abundance of *Panotea* markedly decreased in this study. The abundance of *Panotea* might be inhibited in an acidic environment (pH < 5.40) during anaerobic conditions (McGarvey et al., 2013; Sun et al., 2021). After the fermentation process, the bacterial community structures were diverse in native grass inoculated without or with LAB. The genus *Weissella* dominated the fermentation in the CON group, followed by *Pediococcus* and *Leuconostoc;* whereas, the abundance of *Lactobacillus* was increased and the abundance of *Weissella* was dropped with the increase of LAB species inoculated in the native grass. The previous report indicated that *Lactococcus* and *Pediococcus* initiated the fermentation at the early stage (Cai et al., 1998), and they were replaced by *Lactobacillus* with more acid-tolerant characteristics (Graf et al., 2016). Moreover, the increase of LAB species inoculated in the native grass produced a complex environment and enriched the bacterial diversity. As a consequence, a higher level of *Lactobacillus* was observed in the T3 treatment.

### Correlation analysis of bacterial community and ensiling performance in native grass silage

The variations in fermentation characteristics could be characterized by the diversity of the microbial community (Ni et al., 2017). Moreover, the additives had significant effects on silage quality and microbial community (Wang et al., 2021). In the present study, *Lactobacillus* was negatively associated with pH and NH_3_-N, which is similar to the previous report (Fang et al., 2022). Nevertheless, *Lactobacillus* was also negatively associated with the WSC content, which is in disagreement with the previous study that the genus *Lactobacillus* was positively associated with the WSC content (Fang et al., 2022). In the whole-plant quinoa, the WSC content was higher than 30% of DM and the DM content in the present study was lower than the requirement of 5% of DM. The sufficient WSC content was beneficial for the growth of Lactobacillus and the abundance of *Lactobacillus* was inhibited, while the WSC content is limited. Therefore, the genus *Lactobacillus* was negatively associated with the WSC content. These results provide sufficient data to support that *Lactobacillus* has played a crucial role in improving the fermentation quality and preserving the native grass silage. *Enterobacter* was positively connected with pH and NH_3_-N. Additionally, the genus *Enterococcus* belongs to cocci LAB and could be inhibited in a low pH environment (McGarvey et al., 2013). Therefore, a lower abundance of *Enterobacter* and NH_3_-N content was observed in the T3 treatment.

### Metabolic profile in native grass silage affected by LAB

The predicted functional profiles of the bacterial community were evaluated based on the Kyoto Encyclopedia of Genes and Genomes databases by PICRUSt to assess the metabolic pathways of native grass silage. The first-level directory and second-level directory indicated the first and second metabolic pathway levels, respectively (Wang et al., 2022). A previous report also indicated that multiple sub-directories were involved with various related signal pathways (Ogata et al., 1999). As shown in Figure 3A, metabolism was the primary metabolic pathway, indicating that the bacteria could convert fermentable substrates to various metabolites by the bacterial activities in metabolism pathways. The metabolic pathways with significant differences were including amino acid, carbohydrate, and nucleotide (Figure 3B), which is in accordance with the previous report (Bai et al., 2021). As shown in Figure 3C, carbohydrate metabolism mainly contained gluconeogenesis and glycolysis metabolism, TCA cycles, pentose phosphate pathway, and other pathways (Kanehisa and Goto, 2000). In this study, the carbohydrate metabolism pathway of native grass silage inoculated with LAB was stronger than that of the CON treatment, especially in the T3 treatment. These results indicated that the addition of LAB in native grass silage has a higher capacity to metabolize WSC than the epiphytic bacteria in the control treatment by the addition of LAB which could enhance the competitive strength through the synergistic effects (Svoboda et al., 2018). It was consistent with the previous results that higher LA and AA concentrations were found in the T3 treatment. Additionally, amino acid metabolism is necessary to promote primary metabolism and plant protein synthesis in plants (Wang et al., 2021). In the present study, some of the amino acid metabolism was suppressed after the fermentation process, which could be contributed to the diverse pH environment after ensiling and some undesirable microorganisms that directly influenced the amino acid metabolism were inhibited by the acidic environment (Flythe and Russell, 2004; Wang et al., 2021).

## Conclusion

In conclusion, the present study shows the influences of various LAB on the ensiling performance of native grass by integrating the 16S rRNA gene sequences combined and multiple fermentation parameters. These results showed that LAB could directly affect chemical compositions and fermentation quality by modulating the bacterial community of native grass silage. The complex LAB (*Lactobacillus plantarum, Lactobacillus buchneri*, and *Pediococcus pentosaceus*) exhibited the potential possibility to decrease pH and enhance the relative abundance of LAB in response to obtaining high-quality silage by the synergistic effects. These results suggested that the complex LAB could improve the ensiling performance of native grass silage, and lay a theoretical basis for inoculant application in native grass.

## Data availability statement

The datasets presented in this study can be found in online repositories. The names of the repository/repositories and accession number(s) can be found at: https://www.ncbi.nlm.nih.gov/, PRJNA871931.

## Author contributions

SD and SY designed the study and analyzed the data. SD wrote the manuscript. SD, SY, XJ, YL, and RW performed the experiments. SD, SY, GG, and YJ reviewed and edited the manuscript. YJ funded and supervised the experiments. All authors contributed to the article and approved the submitted version.

## Funding

This work was supported by the Key Program of the National Dairy Innovation Center, China (2021-National Dairy Innovation Center-1).

## Conflict of interest

Author RW is employed by the Inner Mongolia Yihelvjin Agricultural Development Co., Ltd. The remaining authors declare that the research was conducted in the absence of any commercial or financial relationships that could be construed as a potential conflict of interest.

## Publisher's note

All claims expressed in this article are solely those of the authors and do not necessarily represent those of their affiliated organizations, or those of the publisher, the editors and the reviewers. Any product that may be evaluated in this article, or claim that may be made by its manufacturer, is not guaranteed or endorsed by the publisher.
